# Mining of Novel Thermo-Stable Cellulolytic Genes from a Thermophilic Cellulose-Degrading Consortium by Metagenomics

**DOI:** 10.1371/journal.pone.0053779

**Published:** 2013-01-14

**Authors:** Yu Xia, Feng Ju, Herbert H. P. Fang, Tong Zhang

**Affiliations:** Environmental Biotechnology Lab, The University of Hong Kong, Hong Kong SAR, China; J. Craig Venter Institute, United States of America

## Abstract

In this study, metagenomics was applied to characterize the microbial community and to discover carbohydrate-active genes of an enriched thermophilic cellulose-degrading sludge. The 16S analysis showed that the sludge microbiome was dominated by genus of cellulolytic *Clostridium* and methanogenesis *Methanothermobacter.* In order to retrieve genes from the metagenome, *de novo* assembly of the 11,930,760 Illumina 100 bp paired-end reads (totally 1.2 Gb) was carried out. 75% of all reads was utilized in the *de novo assembly*. 31,499 ORFs (Open Reading Frame) with an average length of 852 bp were predicted from the assembly; and 64% of these ORFs were predicted to present full-length genes. Based on the Hidden Markol Model, 253 of the predicted thermo-stable genes were identified as putatively carbohydrate-active. Among them the relative dominance of GH9 (Glycoside Hydrolase) and corresponding CBM3 (Carbohydrate Binding Module) revealed a cellulosome-based attached metabolism of polysaccharide in the thermophilic sludge. The putative carbohydrate-active genes ranged from 20% to 100% amino acid sequence identity to known proteins in NCBI nr database, with half of them showed less than 50% similarity. In addition, the coverage of the genes (in terms of ORFs) identified in the sludge were developed into three clear trends (112×, 29× and 8×) in which 85% of the high coverage trend (112×) mainly consisted of phylum of *Firmicutes* while 49.3% of the 29× trend was affiliated to the phylum of *Chloroflexi*.

## Introduction

Second generation of biofuels derived from lignocellulosic plant biomass represent an important renewable alternative for fossil fuels [1]. Lack of cost-effective technology to overcome the recalcitrant nature of the lignocellulosic substrate impediments its industrial-scale production. Enzymatic deconstruction of plant biomass which could greatly improve lignocellulose hydrolysis with no side-effect of generating fermentation inhibitors was applied as a promising strategy in the popular lignocellulosic biofuel production processes like Simultaneous Saccharification and Fermentation (SSF) or Separate Saccharification and Fermentation (SHF) [2]; nevertheless the relatively low activity of currently available hydrolytic enzymes stands in the way. Thereby retrieving novel effective cellulolytic enzymes from biomass-degrading microbial community is of great potential to boost lignocellulosic biofuel production and the thermo-stable cellulase was especially attractive in this concept for its suitability for industrial application.

Metagenomics, direct analysis of DNA fragments from environmental sample, offers a powerful tool to understand microbial consortium and to discover diverse genes/enzymes in the system. Metagenome-derived cellulase has been successfully identified and isolated from cellulolytic consortia in several studies [3]–[7]. However before the widely introduction of next generation sequencing (NGS) technologies in recent 10 years, metagenomic library construction by cloning was a heavy labor job which suffered from the difficulty in discovery of whole genes. Nowadays with the help of the dramatically increased sequencing depth of NGS, metagenomic had stepped into a new chapter that vast gene mining become literally possible. However, among the various metagenomic studies, a good many of them merely focused on community structure characterization, for example the metagenomic characterization of natural ecosystems like the ocean [8], soil [9], permafrost [10], etc. Although several work had demonstrated great practice in metagenomic gene discovery, for example metagenomic biomass-degrading gene discovery from cow rumen and termite gut[11]–[13], the field of NGS metagenomic gene mining still at its infancy with many potential sources untapped.

In addition, metagenomic projects with NGS technologies are now severely challenging the current computational resources. While not mutually exclusive, there are few alternative methods to ensure coverage completeness of a complicated communities other than enlarging sequencing depth which, due to the giant data set required, may bring up the processing and computational cost to more than a million dollars for a metagenomic project, for instance, it was estimated that a minimum of 6 billion base pairs would be required to obtain the genome sequence of the most dominant population in soil sample, and many times more to obtain genomes from less dominant populations [14]. By contrast, metagenomics of reactors with certain intentionally enhanced functions, for example, enhanced biological phosphorus removal reactor (EBPR), cellulose-degrading reactor, phenol decomposing reactor, sludge digester etc., makes more practical sense for most research institutions lack of such admirable resources, and thus is crucial for wide application of metagenomic techniques. Unfortunately although Albertsen et al. had demonstrated a good example with microbiome in EBPR [15], not much attention had been put in such kind of reactor communities.

As a result, given in mind the application value of novel thermo-stable biomass-degrading enzymes in lignocellulosic biofuel production and the practical power of metagenomic approach in genes mining, in the present study, an effectively enriched thermophilic cellulolytic sludge from a lab-scale methanogenic rector was selected for metagenomic gene mining and community characterization. Functions of different phylotypes within this intentionally enriched microbiome were compared against each other to reveal their individual contribution in cellulose conversion. *De novo* assembly of the metagenome was conducted to discover putative thermo-stable carbohydrate-active genes in the consortia. Additionally, a common flaw in metagenomic analysis only based on either assembled ORFs/contigs or short reads was pointed out and amended by mapping reads to the assembled ORFs.

## Results

### Metagenomic Assembly and Coverage Analysis of the Sludge Metagenome

**Figure 1:**
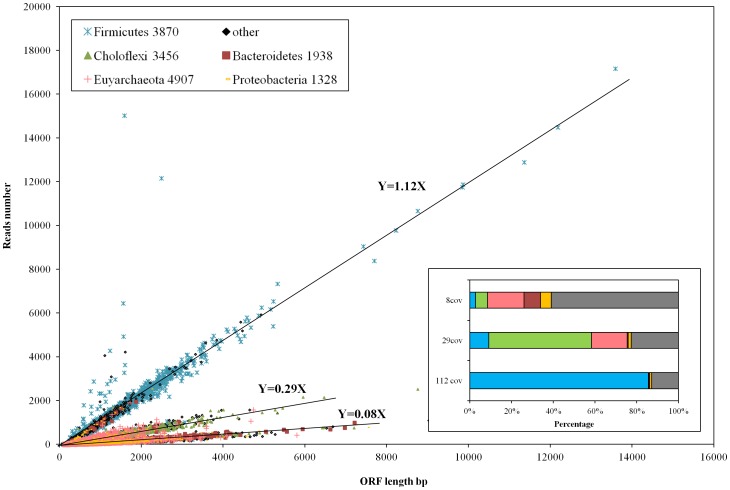
Plot of the number of reads aligned to each ORF as a function of the length of the ORF. The ORFs were generated from contigs longer than 1000 bp. The number of reads aligned to each ORF was determined by SAMTools package. The ORFs were colored according to their taxonomy classification by MEGAN’s LCA algorithm at phylum level. The number of ORFs assigned to each phylum was listed following the phylum name. Insert: taxonomy distribution of ORFs in the three coverage trends demonstrated in the outside frame.

To exploit the metagenome of the enriched thermophilic cellulolytic sludge, short reads generated from the Illumina sequencing was assembled by velvet assembler. Sequences were effectively utilized during the assembly: 75% of the 11,930,760 reads were used in the assembly and 96% of the used reads were assembled into contigs greater than 1 kb, which indicated a sufficient coverage of the metagenome by the current sequencing depth (11.9 million 100 bp reads, total 1.2 Gb; the coverage was further illustrated in Figure 1). The contigs longer than 1 kb were 28.5 Mb in total with N50 of 1141 bp and the largest contigs being 202,468 bp (Table S1). Finally, 31,499 ORFs with an average length of 852 bp were predicted from these contigs; and 64% of these ORFs were predicted to present full-length genes.

The numbers of reads aligned to individual ORFs developed into three distinct coverage trends as shown in Figure 1. The coverage values of the three trends were respectively 112×, 29× and 8× (equals to the product of the slope and the read length of 100 bp). Among the 31,499 defined genes (in term of ORFs), 58.6% of them could be phylogenetically classified at phylum level by the LCA algorithm of MEGAN4 against NCBI *nr* database. Based on the taxonomic classification of ORFs, genes in the high coverage of 112× were largely (85.5%) belong to the phylum of *Firmicutes* while *Choloflexi* took 49.3% of the ORFs in the 29× trend (Figure 1 insert). The phylum of *Euryarchaeota* (4907 ORFs) evenly distributed in the lower coverage trends of respectively 17.5% in 29× trend and 17.0% in the 8× trend (Figure 1 insert). Unlike the even distribution of *Euryarchaeota*, the major proportion of *Firmicutes* (72% of 3870 ORFs) was fitted into the higher coverage trend (112×). In addition, even under the coverage as high as 112×, it still had 12.8% of the ORFs longer than 1kb could not be phylogenetically assigned into any known phylum which revealed our limited understanding of the microbial world, even for some dominant populations in this enriched simple microbial community.

### Community Structure of the Sludge Metagenome Based on 16S/18S rRNA Genes

Three different databases of 16S/18S rRNA genes, i.e. Silva SSU, RDP and Greengenes, were used to determine community structure via MG-RAST at E-value cutoff of 1E-20. A major agreement was followed by the three databases that 16S/18S rRNA gene occupied around 0.15% of the total metagenomic reads. According to Silva SSU, 83.4% of the rRNA sequences affiliated to *Bacteria*, 11.1% to *Archaea*, 1.3% to *Eukaryota*, 0.3% to virus and 4.0% unable to be assigned at domain level. *Clostridium*, taking 55% of the population, was the major cellulose degraders in the sludge microbiome, while the methanogens in the sludge consortium were belong to the genus of *Methanothermobacter* and *Methanosarcina* which accounted for respectively 11.2% and 1.3% of the microbial population (Figure S1). A rarefaction curve was drawn by MEGAN with the 16S/18S reads from the metagenomic dataset. Satisfactory coverage of the reactor microbiome was illustrated in the rarefaction curve that the curve already passed the steep region and leveled off to where fewer new species could be found when enlarged sequencing depth (Figure S2).

### Phylogenetic Analysis of the Sludge Metagenome Based on Protein Coding Regions

**Figure 2:**
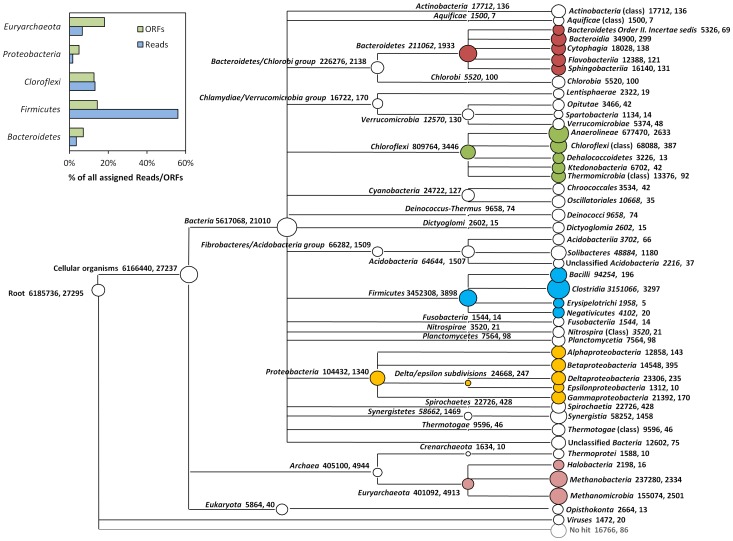
Taxonomy classification of the metagenome at class level based on RMORF approach. ORFs were assigned by default MEGAN LCA algorithm; only nodes with over 5 ORFs and 1000 reads assigned are shown. The circles are drawn based on the number of reads assigned to the particular node. The number after description denotes, respectively, the sum of reads and ORFs assigned below the particular node. The circles are colored according to its classification at phylum level as in Figure 1. Insert: the relative distribution of annotated reads and ORFs in the major phyla.

Besides reads analysis based on 16S rRNA gene, community structure of the sludge metagenome was further studied based on the protein coding regions. Both the reads and assembled ORFs were used in this approach: Reads were annotated via the MG-RAST online sever against GenBank database with E-value cutoff of 1E-5 while Annotation of ORF was carried out by blast against NCBI *nr* database at E-value cutoff of 1E-5. It’s interesting to notice that the community structure revealed by ORFs annotation were noticeably inconsistent with annotation based on reads. For example, Phylum *Firmicutes* taken relative small proportion (14%) of the annotated ORFs evidently dominated the reads distribution by taking 55% of the annotated reads (Figure 2 insert). The correlation coefficient between community structure at phylum level revealed by reads and ORFs annotation was as low as 0.4. Furthermore the read annotation were somewhat problematic for its low annotation efficiency that only less than 10% of the 11,930,760 pair-end reads could be annotated.

With in mind the defects of individual reads and ORFs annotation, a method combining these two approaches was applied at last. ORFs were firstly annotated as mentioned above and then the 11,930,760 pair-end reads were aligned to the ORFs for accurate quantification. Taxonomy classification based on this combined annotation method was in consistency with classification based on 16S/18S in the taxon down to order level (Figure S3) with an exception that the class *Anaerolineae* occupying 14.6% of the BLAST-annotated reads was not found in the 16S/18S annotation (Figure S3a). The dominant classes of the thermophilic consortia included (listed quantitatively): *Clostridia* (3151066 reads, 3297 ORFs), *Anaerolineae* (677470 reads, 2633 ORFs), *Methanobacteria* (237280 reads, 2334 ORFs) and *Methanomicrobia* (155074 reads, 2501 ORFs) (Figure 2).

### Functional Analysis

930,939 reads was annotated by the SEED subsystem in MG-RAST server at E-value cutoff of 1E-5; their annotation revealed a confined functional (584 of 1519 possible functions in Subsystems) and taxonomic (detection of 421 putative GenBank taxa) diversity in the reactor sludge metagenome. Relative abundance of the SEED subsystems was shown in Figure S4.

Figure S5 demonstrated the comparison of *Bacteria* and *Archaea* in the SEED subsystems on Carbohydrate metabolism (Figure S5) and One-carbon metabolism (Figure S5 insert). The number of reads assigned to a specific subsystem (primary y axis) indicates the relative contribution of the domain in the corresponding function category, whereas the percentage of reads assigned (secondary y axis) represents the domain’s preference to the functional category. As shown in Figure S5, considering the evident dominance of *Bacteria* in the community, it is not surprising to find that *Bacteria* played an important role in all subsystems involved in carbohydrate metabolism except for the one-carbon metabolism in which *Archaea* was crucial as it exclusively contributed to the methanogenesis process (Figure S5 insert). Additionally, the functions of genera *Clostridium* and *Thermoanaerbacterium* were studied in the same manner. *Clostridium* in the sludge metagenome showed stronger selection in degrading polysaccharides and di-and oligosaccharides while *Thermoanaerobacterium* preferred more on metabolizing monosaccharides (Figure S6).

**Figure 3:**
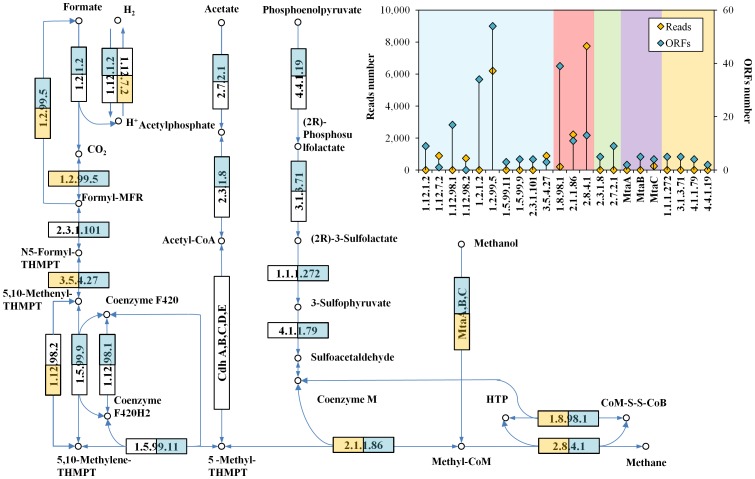
ORF and Reads assignment to KEGG Methanogenesis Pathway. Blue square indicates this enzyme has at least one ORF assigned; Yellow square indicates this enzyme has at least one read assigned. Insert: numbers of ORFs and reads assigned to enzymes in the pathway. Metabolism modules are highlighted in different colors: blue, “Formate to Methane”; green, “Acetate to Methane”; purple, “Methanol to Methane”; yellow, “Coenzyme M synthesis”; red, enzymes shared among different modules.

The KEGG methanogenesis modules were shown in Figure 3; the complete pathway of “Format/Hydrogen/CO_2_ to methane” and “Methanol to methane” was revealed in the consortia while the acetyl-CoA decarbonylase/synthase complex subunit alpha [EC:1.2.99.2] (shown as box of “Cdh, A,B,C,D,E” in Figure 3) was not observed indicating unfavorable “Acetate to methane” process of the consortia. In addition, the high proportion of formate dehydrogenase [EC:1.2.1.2] and formylmethanofuran dehydrogenase subunit A [EC:1.2.99.5] (Figure 3 insert) pointed out a more active metabolizing of formate/hydrogen/CO_2_ to methane in the thermophilic sludge consortium. Comparing to the functional annotation by assembled ORFs, many key enzymes in the methane production process especially the enzymes involved in the “Co-enzyme M synthesis module” was missing in the read annotation, indicating that short reads was not suitable for functional analysis of metagenome due to the low annotation efficiency.

### Mining of Thermo-stable Carbohydrate-active Genes in the Sludge Metagenome

To identify candidate carbohydrate-active genes from the sludge metagenome, we performed *de novo* assembly and predicted 31,499 ORFs with an average length of 852 bp and 64% of the 31,499 ORFs were predicted to represent full-length genes. To examine the validity of the de novo assembly, we experimentally testified a random subset of 10 putative carbohydrate-active genes (length from 98 to 917 Amino Acids (AA)). The target gene fragments were amplified by specifically designed primers with the DNA extract used to generate the metagenomic data as PCR template. Using single set of PCR condition, we obtained 9 out of 10 candidate genes (90%) with the predicted size (Figure S7). For further validation, the PCR product was sequenced. The sequenced PCR products showed >99% ungapped sequence identity to the computationally predicted putative genes (Table S2).

To find out the carbohydrate-active genes in the predicted gene pool, the ORFs were firstly searched against the PfamA database based on the Hidden Markol Model (HMM) at E-value cutoff of 1E-4 [11]. The searching results against PfamA database was further screened against the CAZy database for candidate carbohydrate-active genes. Only those CAZy families having clear Pfam models were counted to ensure the accuracy of gene mining (Table S3 and S4). 253 candidate genes were identified with a significant match to at least one relevant glycoside hydrolase domain or carbohydrate-binding module as classified in the CAZy database (Table S3 and S4). The candidate genes found in the enrich sludge metagenome fell into a variety of CAZy families (30 out of 130 GH families and 5 out of 64 CBM families defined in the CAZy database). The major GH families were GH3, GH2 and GH9, respectively taking 17.4%, 16.7% and 13.8% of the total annotated genes in GH families, while CBM3 and CBM6 each took 38.6% of genes belongs to CBM families (Table S3 and S4).

**Figure 4:**
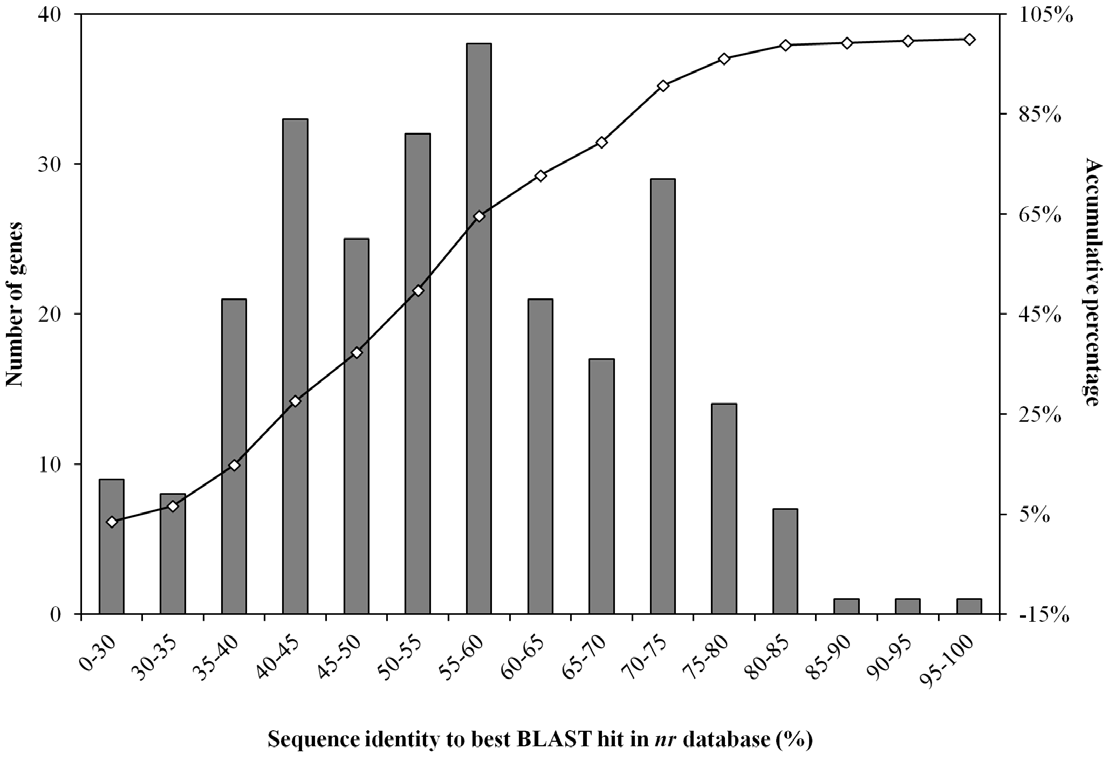
Similarity distribution of predicted ORFs with thermo-stable carbohydrate-active genes against NCBI *nr* database by BLASTp (E-value ≤1E-5).

The retrieved genes were then blast against the NCBI *nr* database to found out their similarities to known genes. The results showed that around half of the predicted thermophilic cellulolytic genes in the sludge metagenome had quite low (less than 50%) similarity to known genes in *nr* database (Figure 4). This poor demonstration of retrieved genes in comprehensive database like *nr* indicated a high potential of existence of novel thermo-stable genes in the sludge metagenome.

## Discussion

### Metagenomic Assembly and Coverage Analysis of the Sludge Metagenome

Adequate coverage is critical for the flawless understanding of a metagenome. Hess et al. (2011) had showed a satisfying coverage of a cow rumen metagenome with 65% of the 267.9 Gb Illumina data set used in the assembly (Table S5). Delmont et al. estimated a data size of 120.1 Gb of 454 sequences (equivalent to 405 Titanium runs) to fully cover the grassland soil metagenome [9]. Not mentioning the high sequencing and processing cost, the huge metagenome dataset required to cover such complex metagenome inevitably proscribed the application of this technique within several countable top institutions with the super computational capacity. Nevertheless the present study investigating an enriched reactor microbiome with a compact scale of dataset (1.2 Gb), may demonstrate an applicable practice of metagenomic technology which could be referred to in common research conditions.

Similar to the coverage trends illustrated in Figure 1, two coverage trends were observed in a recent study of soil metagenome [9]. Delmont et al. stated that *Proteobacteria* genomes from the lower coverage trend might be assembled more rapidly than *Firmicutes* and *Verrucomicrobia* in the higher coverage trend. Correspondingly, the phylogenetic annotation of the three coverage trends in this study revealed that genomes of *Choloflexi* and *Euryarchaeota* might be assembled more effectively than those from *Firmicutes* who took 85.5% of the 112× trend (Figure 1). It was supposed by Delmont et al. the presence of regions that limit assembly (for example, insertion sequences regions) and the complexity of diversity among taxa was part of the reasons for the higher coverage requirement of genomes in the high coverage trend. However to the opposite of their claim, the harder-to-assembled *Firmicutes* showed a more uniform phylogenetic structure than *Euryarchaeota* in the lower trend. The major proportion of *Firmicutes* (72% of 3870 ORFs) was fitted into the higher coverage trend (112×), whereas *Euryarchaeota* evenly distributed in the 29× trend and the 8× trend. Our previous 16S rRNA gene analysis results also demonstrated the simple structure under phylum *Firmicutes* that *Clostridium*, the major cellulose degrader, had taken over 95% of the phylum [16]. Thus the complexity of diversity among taxa might not contribute to the coverage scattering in this study, instead the clear dominance of phylum *Firmicutes* was in good part responsible for its high coverage and the relatively limited assembly efficiency because the velvet assembler showed a coverage saturation at around 30–40× coverage that further increasing read coverage over 30–40× could not improve assembly in terms of N50 and length of longest contig [17].

### Phylogenetic Analysis of the Sludge Metagenome Based on Protein Coding Regions

It is important to make function-based phylogenetic assignment in order to understand the functional contribution of different taxonomy units in the metagenome. However such approach based on short reads has a shortage of low annotation efficiency at certain annotation accuracy like in this study only less than 10% reads were annotated by MG-RAST comparison against SEED subsystem at E-value cutoff of 1E-5. In addition, reads annotation may result in overlook of some important functional information, for example, the “Co-enzyme M synthesis” which is the functional core of methanogenesis was completed undetectable by short reads annotation in the present study (Figure 3). On another hand, the annotation based on assembled results, such as contigs or ORFs, was hardly representative because the information of reads coverage of the contigs/ORFs was not counted in such quantification. For a metagenome with scattering coverage like the enriched consortia used in the present study (Figure 1), incorrect taxa distribution would be easily resulted based on annotation of ORFs (Figure 2 insert). Such annotation inconsistency between reads and ORFs was also found in the metagenome of the grassland soil [9]. However, due to the high computational cost of direct phylogenetic annotation of protein coding reads, assemblies like contigs/ORFs were still used for phylogenetic quantification in many studies, for example the metagenomic characterization of EBPR by Albertsen et al. [15], which apparently requires some sort of correction.

In order to correct the ORFs annotation as well as to overcome the defects of reads annotation, an alternative method based on the annotation of ORFs and mapping reads to ORFs was applied in the present study. The result of this combined method was in consistency with classification based on 16S/18S in the taxon down to order level (Figure S3). The discrepancy at lower levels of family and genus might be in part explained by the unavoidable phylogenetic ambiguity of functional genes. About 39.8% of the reads were assigned by this method, which was 4 times higher than the direct taxonomic annotation of short reads.

### Functional Analysis

It is not surprising to find that more functional information could be covered by the assembly results like ORFs, for example the “acetate to methane” and “coenzyme M synthesis” modules which were undetectable by short reads, were revealed in ORF annotation (Figure 3). However, since the current version of MEGAN software package was unable to parse the reads to ORFs alignment result into functional systems like SEED subsystem or KEGG pathway, the functional comparison between different taxonomic units showed below was based on the direct annotation of short reads using MG-RAST at E-value cutoff of 1E-5.

Cooperation between *Bacteria* and *Archaea* was demonstrated in the metagenome that *Bacteria* initiated metabolism of cellulose by converting the polysaccharide and resulted oligosaccharides (Polysaccharides and Di- and oligosaccharides metabolism, Figure S4) into mono-sugars which could enter the central carbohydrate metabolism where via glycolysis to release energy to the consortium as well as provide NADH (Nicotinamide adenine dinucleotide) for the following anaerobic fermentation, while methanogens (main part of *Archaea*) further anaerobically oxidize fermentation intermediates to methane (Methanogenesis, Figure S4 insert) to achieve final oxidation of carbohydrate and remove inhibitory products for *Bacteria* metabolism (Figure S4).

Both genera of *Clostridium* and *Thermoanaerobacterium* had been reported to be able to metabolize lignocellulosic feedstock [2], however, our previous study found that growth of *Thermoanaerobacterium* over *Clostridium* under acidic condition (pH <6.0) will significantly reduce the cellulose degrading capacity of the consortia [16]. This phenomenon could be explained by the results shown in Figure S6 that genus *Thermoanaerobacterium* of the sludge metagenome displayed deficient capacity towards polysaccharides, and Di- and oligosaccharides metabolism comparing to *Clostridium*.

### Mining of Thermo-stable Carbohydrate-active Genes in the Sludge Metagenome

Lignocellulose degradation requires a broad array of enzymes and associated proteins. Most of the enzymes involved in the process are GH (glycoside hydrolase) families which hydrolyze the glycosidic bond between carbohydrates or between a carbohydrate and a non-carbohydrate moiety [18]. Additionally, the CBMs, bringing the biocatalyst into intimate and prolonged association with its recalcitrant substrate, determine the rate of catalysis [2]. Therefore, the present study mainly focused on the GH families and CBM families. The CAZy database maintains updated information on GH families and CBM families according to their classifications of amino acid sequences similarity. Currently there are 130 GH families and 64 CBM families. The searching results by HMM based on PfamA database was further screened against the CAZy database for candidate carbohydrate-active genes (Table S3 and S4). Glycoside hydrolase (GH) families are assigned to different categories based on the classification published by Pope et al. [19].

**Figure 5:**
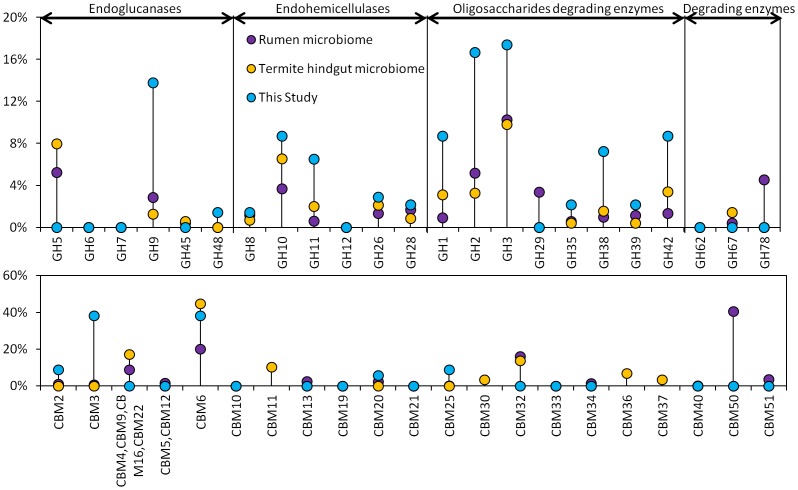
Comparison of predicted carbohydrate-active genes (top chart) and carbohydrate-binding modules (bottom chart) in three cellulosic materials fed metagenomes: rumen microbiome [**11**] **, termite hindgut microbiome** [**12**] **and the enriched thermophilic cellulolytic sludge microbiome from this study.** Glycoside hydrolase (GH) families are assigned to different categories based on the classification published by Pope et al. [19] PFAMs associated with particular GHs and CBMs are listed in Table S3 and S4. Gene counts include both complete ORFs and ORF fragments.

It is interesting to notice that: first, there is a wide diversity of GH catalytic modules in the thermophilic sludge microbiome, indicated by the 236 modules belonging to 30 GH families, which was comparable to bovine rumen with 35 GH families [20]. But to great contrast, only 16 carbohydrate-binding modules from 5 families (CBM2, CBM3, CBM6, CBM20 and CBM25) were observed (Table S4). Comparing to rumen [11] and termite gut microbiomes [12], the high fraction of CBM3, a common component of cellulosomes [2] (Figure 5), indicated a thermo-stable cellulosome-based metabolism system, in which initial attachment of the microorganisms to the recalcitrant substrate surface played a critical role in the sludge metagenome. Nearly all of the CBMs were found in ORFs affiliated to *Firmicutes* (31 out of 33 CBMs), which was probably resulted from the cellulosome based attached growth model adopted by the dominant cellulolytic *Clostridium* strains under that phylum. For the GH families, most of the GH genes were *Bacteria* originated (116 out of 236 GHs), while 12 were assigned as *Archaea*. However, around half of the GH families (108 out of 236 GHs) came from the ORFs which were unable to be assigned to any known phylum in the NCBI *nr* database at E-value cutoff of 1E-5, demonstrating that many of the thermo-stable carbohydrate-active genes in the sludge were contributed from the populations which were not well phylogenetically characterized. Comparing to other two mesophilic plant fed microbiomes [11], [12], the thermophilic sludge metagenome showed high proportion of endoglucanases as GH9 (13.8% of GH families, Table S3) whose C-terminus catalytic domain usually has rigidly attached a CBM3 family [21]. The dominance of GH9 and CBM3 in the thermophilic sludge metagenome indicated a beneficial thermo-stable cellulosome based polysaccharide metabolism pathway as compared to mesophilic system of rumen and termite gut [11], [12] (Figure 5). More importantly, a round half of the thermophilic cellulolytic genes identified in the sludge metagenome had less than 50% similarity to known genes in *nr* database (Figure 4), indicating the possible existence of novel thermo-stable genes which had never be identified elsewhere. Further experiments are undergoing to validate the cellulose-degrading activity and thermo-stability of these predicted genes from the sludge metagenome. Apart from enzymes devoted to the hydrolysis of the main chain of cellulose (GH5, GH9), hemicellulose (GH10, GH11), and pectins (GH28), the sludge metagenome displayed a larger diversity of enzymes that digested the side chains of these polymers and oligosaccharides thereof (Figure 5). The families GH2 and GH3, which contain a large range of glycosidases were particularly abundant, with >34% of GH families (Table S3).

## Materials and Methods

### Thermophilic Anaerobic Cellulolytic Sludge

Anaerobic digestion sludge (collected from Shek Wu Hui wastewater treatment plant Hong Kong SAR, China) was used to enrich cellulolytic methanogenic consortia in a sequential batch reactor (SBR) for two years at 55°C and pH >6.0 by feeding microcrystalline cellulose as substrate and glucose as co-substrate at COD ratio of 10∶1. Our previous study showed that the enriched sludge had a volatile suspended solid (VSS) of 1.4 g/l and was capable to convert cellulose at 1.15 kg cellulose m^−3^ d^−1^ [16].

### DNA Extraction

Genomic DNA was extracted from 500 mg sample with FastDNA SPIN Kit for Soil (MP Biomedicals, LLC, Illkirch, France). The exacted DNA was 186 ng/µl and had a 260/280 ratio of 1.89 (Nanodrop, ND-1000). DNA library of ∼180 bp was prepared following the manufacturer’s instruction (Illumina). Briefly, DNA fragmentation was carried out by Covaris S2 (Covaris, 01801-1721). The flow cell was sequenced by BGI (Shenzhen, China) using Illumina HiSeq2000 technology generating 2×100 bp paired-end reads. The base-calling pipeline (version Illumina Pipeline-0.3) was used to process the raw fluorescence images and call sequences.

### Metagenome Assembly and Coverage Statistics

The paired-end sequences were quality-checked by discarding any read containing ambiguous base of letter N and then trimming off the sequencing adaptors to get reads of 100 bp in length. Reads after quality control (11,930,760 reads, 1.2 Gb) had been submitted to NCBI SRA database under accession number of SRA057365. Reads after quality check were assembled using velvet (version 0.7.62) [17] by options: k = 31, cov_cutoff = 3, and exp_cov = 14. Only contigs longer than 1 kb were used in the following analysis [11]. The read coverage of each ORF/contig was quantified via aligning the paired-end reads to ORFs/contigs by Bowtie (0.12.7) [22]. For each read, only the best alignment was kept, allowing up to 2 mismatches over the entire length (Bowtie options: -k 1 -f –v 2–best –pairtries 200–chunkmbs 50000). Gap was not allowed during the alignment. And the statistics of alignment coverage was carried out by the SAMtools package [23].

The assembled contigs longer than 1 kb were subject to gene prediction using online MetaGeneMark [24] under Kingdom of mixture of *Bacteria* and *Archaea* with 6-LBA model (Di-codon frequencies fit by logistic regressions on GC content). Totally 31,499 open reading frames were defined by the MetaGeneMark tool.

### Validation of Metagenome Assembly

To verify the accuracy of assembly, a random subset of 10 assembled candidate genes were selected to design specific primer sets. PCR (polymerase chain reaction) were conducted using the designed primers, respectively, with the same extracted DNA as the template. The PCR condition was as follow: an initial 5 min denaturation at 94°C, 35 cycles of 1 min at 94°C and 3.5 min at 68°C, and a final 5 min extension at 68°C. Each PCR volume contained 50 ng DNA, 200 nM of each primer (Integrated DNA Technologies, US), 25 µL 2 × Premix Ex Taq (TaKaRa, China), and ddH_2_O up to 50 µL. If the determined sizes of the PCR products matched the sizes of the assembled genes, the purified product (PureLink™ PCR Purification Kit, Invitrogen, US) was sent out for Sanger sequencing (GRC, The University of Hong Kong). Genes longer than 400 AA were sequenced from both forward and reverse ends.

### Carbohydrate-active Gene Prediction

Next, amino acid sequences of the predicted ORFs were screened against PfamA database version 26.0 [25] by Pfam_scan (E-value cutoff of 1E-4) [26] for particular glycoside hydrolase (GH) families and carbohydrate binding module (CBM) as classified by the CAZy (Carbohydrate Active enZyme) database [27].

### Taxonomy and Functional Annotation

Reads passed primary quality control at BGI (11.9 million reads, 1.2 Gb) were submitted to the MG-RAST server (v 3.0) [28] for taxonomic and functional annotation. The default quality control pipeline (QC pipeline) of MG-RAST was used to remove technological duplicates resulted from sequencing bias. Taxonomic annotation based on 16S/18S rRNA genes was performed against rRNA gene databases of RDP, Silva SSU and Greengenes using E-value cutoff of 1E-20 [10], while taxonomy of protein-coding reads was performed against GenBank with E-value cutoff of 1E-5. Functional annotation was conducted against SEED subsystem and KEGG database using E-value cutoff of 1E-5 and hierarchical classification algorithm.

The predicted ORFs were subject to BLAST [29] search against NCBI *nr* database using E-value cutoff of 1E-5, num_alignments 50 and num_descriptions 50 before being assigned to various taxonomy and functional units using the lowest common ancestor (LCA) algorithm with default parameters by MEGAN4.0 [30]. In addition to guarantee annotation accuracy of ORFs, the LCA algorithm was applied to avoid the influence of chimera, as any chimeric ORF with contradictory annotation will be discarded in the LCA assignment. The distribution, as the percentage of reads assigned to an item in the total number of annotated sequences for each database or annotation method, was used for comparison.

## Supporting Information

Figure S1 **Relative distribution of microbial genera (in percentage of the total annotated reads) in the enriched thermophilic cellulolytic sludge metagenome.**
(DOC)

Figure S2 **Rarefaction curve derived from the 16S/18S reads from the metagenome.**
(DOC)

Figure S3 **Relative reads distribution (in percentage of reads annotated) among major taxonomy levels annotated by two independent methods: white bar: based on reads aligned to ORFs classified by blast against NCBI nr database; Gray bar: based on reads annotated by MG-RAST using Silva SSU database.** Chart a, b, c and d respectively represents the Class, Order, Family and Genus levels.
(DOC)

Figure S4 **Relative Abundance of SEED subsystems.** Percentage of each subsystem was shown above the corresponding bar.
(DOC)

Figure S5 **Relative distribution of different metabolism subsystems of Archaea and Bacteria in the enriched thermophilic cellulolytic consortia using SEED subsystems in the MG-RAST server.** Outside: Carbohydrates Metabolism (Level 2 subsystem); Insert: One-carbon Metabolism (Level 3 subsystem).
(DOC)

Figure S6 **Relative distribution of different metabolism subsystems of genus** ***Clostridium*** **and** ***Thermoanaerobacterium*** **in the enriched thermophilic cellulolytic sludge metagenome using SEED Carbohydrates Metabolism subsystems in the MG-RAST server.**
(DOC)

Figure S7 **Gel analysis results of the predicted putative genes.** Gene size of each band was shown in unit of amino acids. The incorrect gene size was marked in red frame.
(DOC)

Table S1 **Velvet assembly statistics.**
(DOC)

Table S2 **Properties of the 10 predicted carbohydrate-active enzyme candidates tested for assembly authority.**
(DOC)

Table S3 **Glycoside hydrolases from the enriched thermophilic cellulolytic culture.**
(DOC)

Table S4 **Carbohydrate binding modules from enriched thermophilic cellulolytic culture.**
(DOC)

Table S5 **Comparison between metagenomic study of cow rumen microbes (10) and this study.**
(DOC)
