# Supplementary material for: Mining of Novel Thermo-Stable Cellulolytic Genes from a Thermophilic Cellulose-Degrading Consortium by Metagenomics
Source: PLoS One. 2013 Jan 14;8(1):e53779. doi: 10.1371/journal.pone.0053779 (PMC3544849; doi:10.1371/journal.pone.0053779)
Supplement: Figure S1 — Relative distribution of microbial genera (in percentage of the total annotated reads) in the enriched thermophilic cellulolytic sludge metagenome. (DOC) [file pone.0053779.s001.doc]

Figure S1 Relative distribution of microbial genera (in percentage of the total annotated reads) in the enriched thermophilic cellulolytic sludge metagenome.
