# Supplementary figures and images for: Mining of Novel Thermo-Stable Cellulolytic Genes from a Thermophilic Cellulose-Degrading Consortium by Metagenomics
Source: PLoS One. 2013 Jan 14;8(1):e53779. doi: 10.1371/journal.pone.0053779 (PMC3544849; doi:10.1371/journal.pone.0053779)

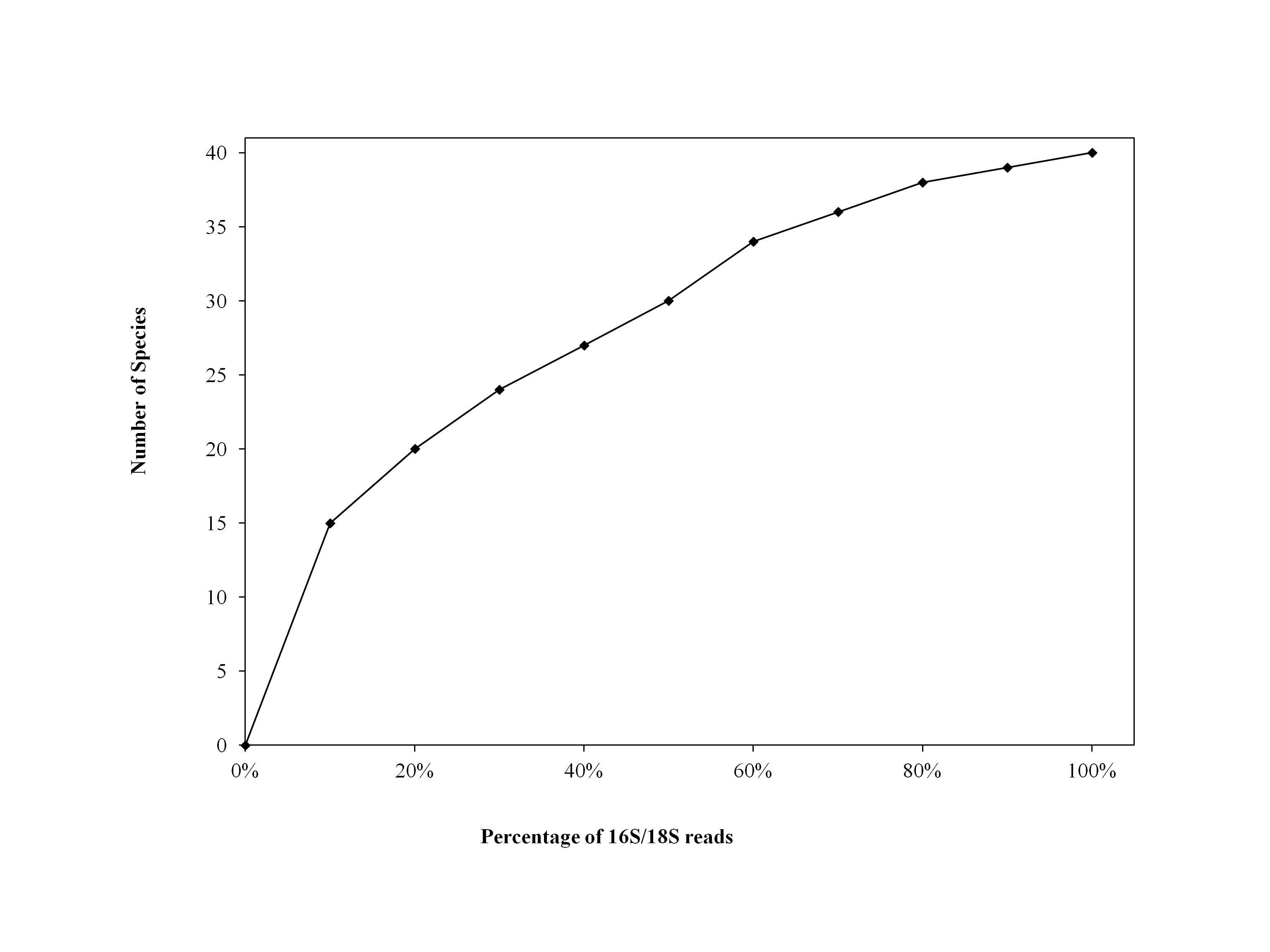


Figure S2 Rarefaction curve derived from the 16S/18S reads from the metagenome

Supplement: Figure S2 — Rarefaction curve derived from the 16S/18S reads from the metagenome. (DOC) [file pone.0053779.s002.doc]
