# Supplementary material for: Mining of Novel Thermo-Stable Cellulolytic Genes from a Thermophilic Cellulose-Degrading Consortium by Metagenomics
Source: PLoS One. 2013 Jan 14;8(1):e53779. doi: 10.1371/journal.pone.0053779 (PMC3544849; doi:10.1371/journal.pone.0053779)
Supplement: Figure S3 — Relative reads distribution (in percentage of reads annotated) among major taxonomy levels annotated by two independent methods: white bar: based on reads aligned to ORFs classified by blast against NCBI nr database; Gray bar: based on reads annotated by MG-RAST using Silva SSU database. Chart a, b, c and d respectively represents the Class, Order, Family and Genus levels. (DOC) [file pone.0053779.s003.doc]

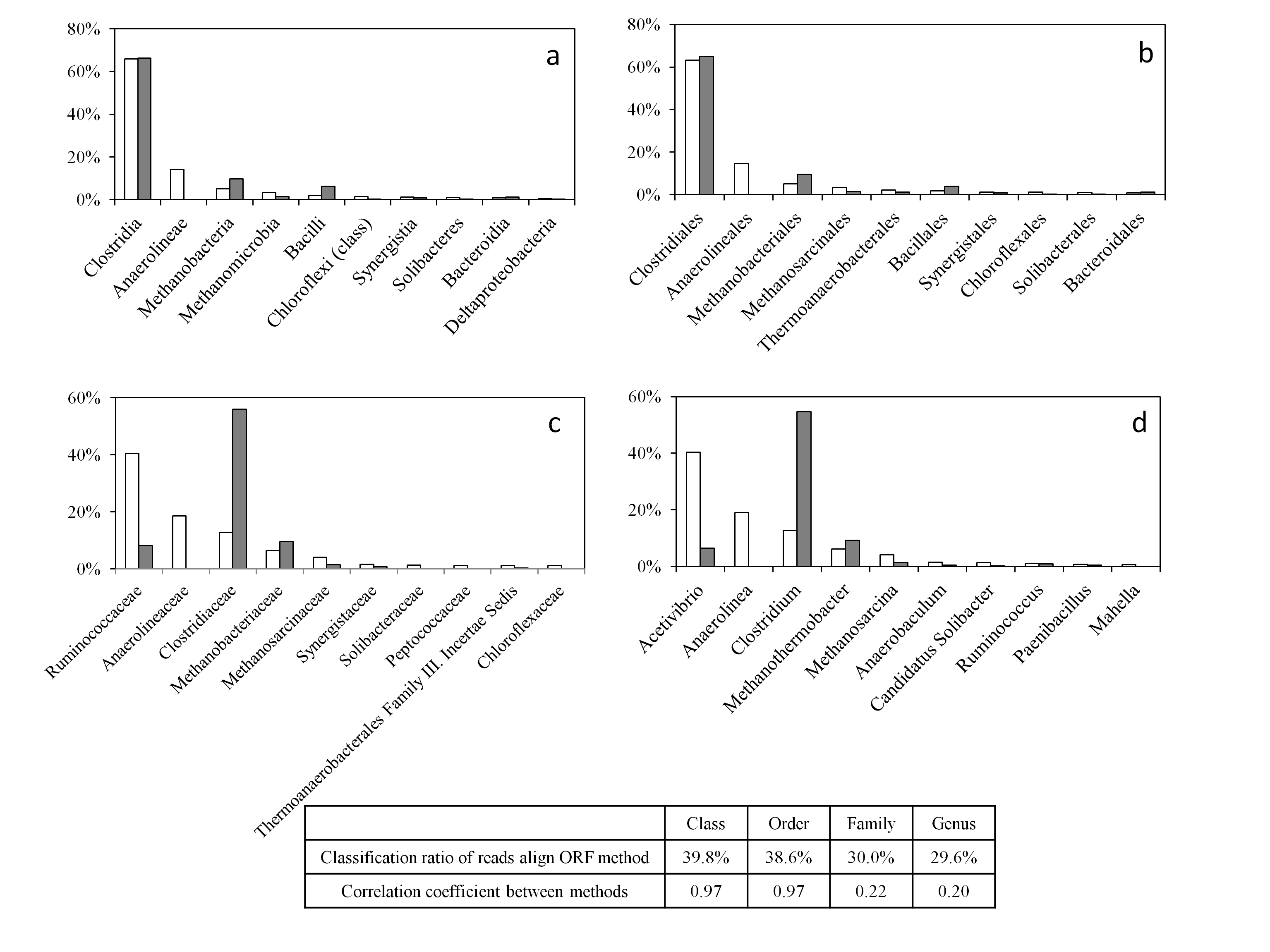


Figure S3 Relative reads distribution (in percentage of reads annotated) among major taxonomy levels annotated by two independent methods: white bar: based on reads aligned to ORFs classified by blast against NCBI nr database; Gray bar: based on reads annotated by MG-RAST using Silva SSU database. Chart a, b, c and d respectively represents the Class, Order, Family and Genus levels.
