# Supplementary material for: Mining of Novel Thermo-Stable Cellulolytic Genes from a Thermophilic Cellulose-Degrading Consortium by Metagenomics
Source: PLoS One. 2013 Jan 14;8(1):e53779. doi: 10.1371/journal.pone.0053779 (PMC3544849; doi:10.1371/journal.pone.0053779)
Supplement: Figure S4 — Relative Abundance of SEED subsystems. Percentage of each subsystem was shown above the corresponding bar. (DOC) [file pone.0053779.s004.doc]

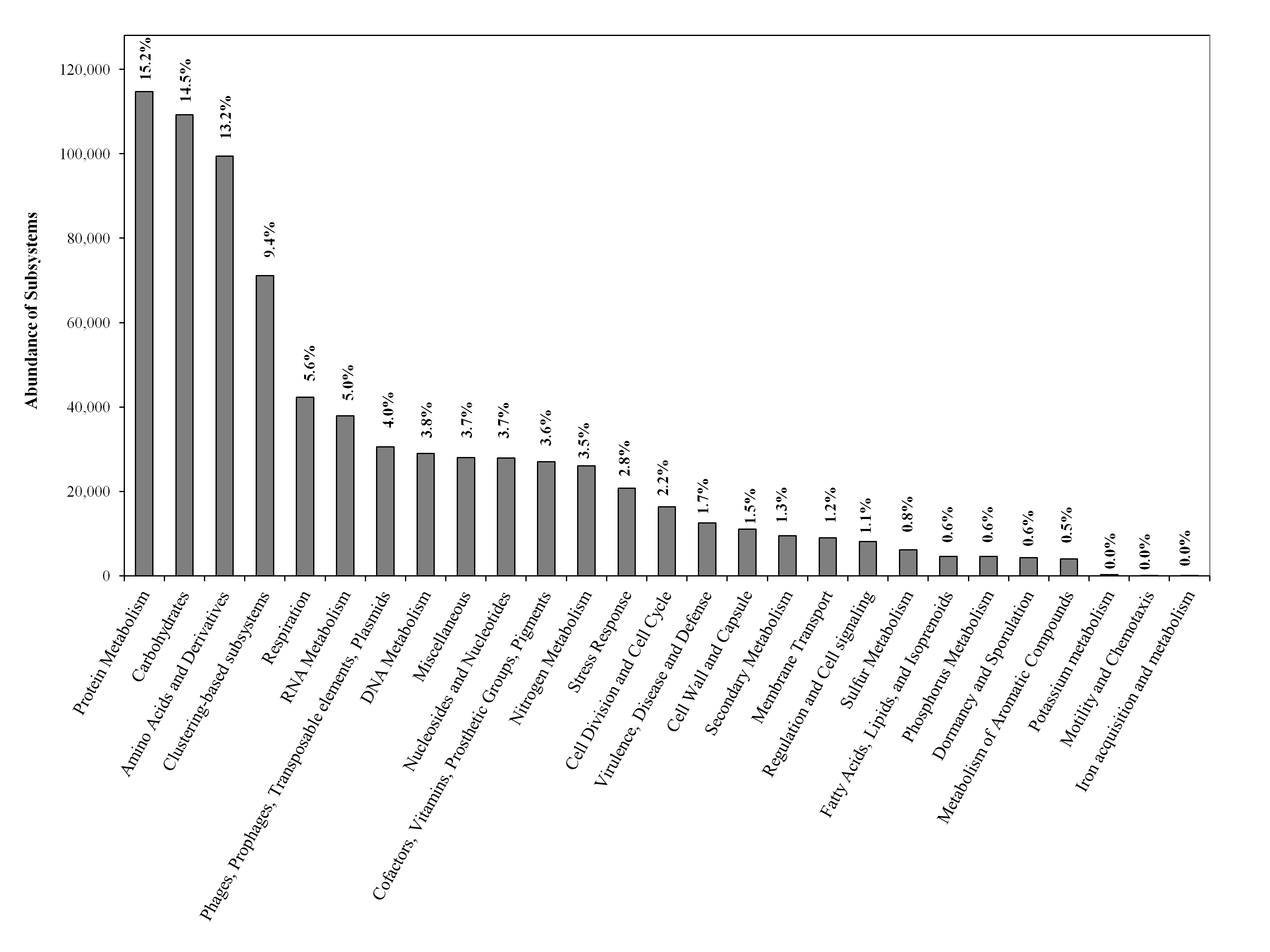


Figure S4 Relative Abundance of SEED subsystems. Percentage of each subsystem was shown above the corresponding bar.
