# Supplementary material for: Mining of Novel Thermo-Stable Cellulolytic Genes from a Thermophilic Cellulose-Degrading Consortium by Metagenomics
Source: PLoS One. 2013 Jan 14;8(1):e53779. doi: 10.1371/journal.pone.0053779 (PMC3544849; doi:10.1371/journal.pone.0053779)
Supplement: Figure S5 — Relative distribution of different metabolism subsystems of Archaea and Bacteria in the enriched thermophilic cellulolytic consortia using SEED subsystems in the MG-RAST server. Outside: Carbohydrates Metabolism (Level 2 subsystem); Insert: One-carbon Metabolism (Level 3 subsystem). (DOC) [file pone.0053779.s005.doc]

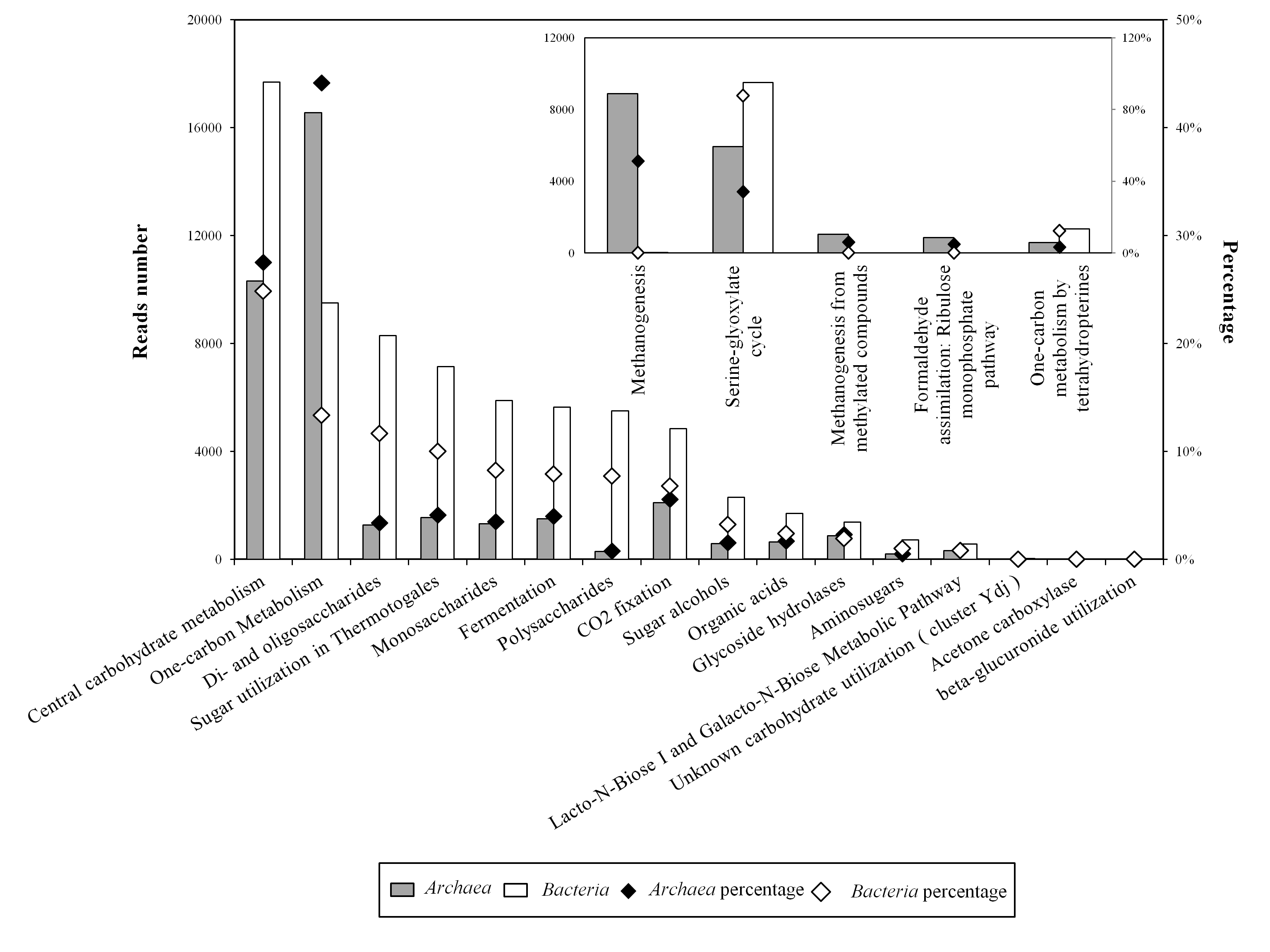


Figure S5 Relative distribution of different metabolism subsystems of *Archaea* and *Bacteria* in the enriched thermophilic cellulolytic consortia using SEED subsystems in the MG-RAST server. Outside: Carbohydrates Metabolism (Level 2 subsystem); Insert: One-carbon Metabolism (Level 3 subsystem).
