# Supplementary material for: Mining of Novel Thermo-Stable Cellulolytic Genes from a Thermophilic Cellulose-Degrading Consortium by Metagenomics
Source: PLoS One. 2013 Jan 14;8(1):e53779. doi: 10.1371/journal.pone.0053779 (PMC3544849; doi:10.1371/journal.pone.0053779)
Supplement: Figure S6 — Relative distribution of different metabolism subsystems of genus Clostridium and Thermoanaerobacterium in the enriched thermophilic cellulolytic sludge metagenome using SEED Carbohydrates Metabolism subsystems in the MG-RAST server. (DOC) [file pone.0053779.s006.doc]

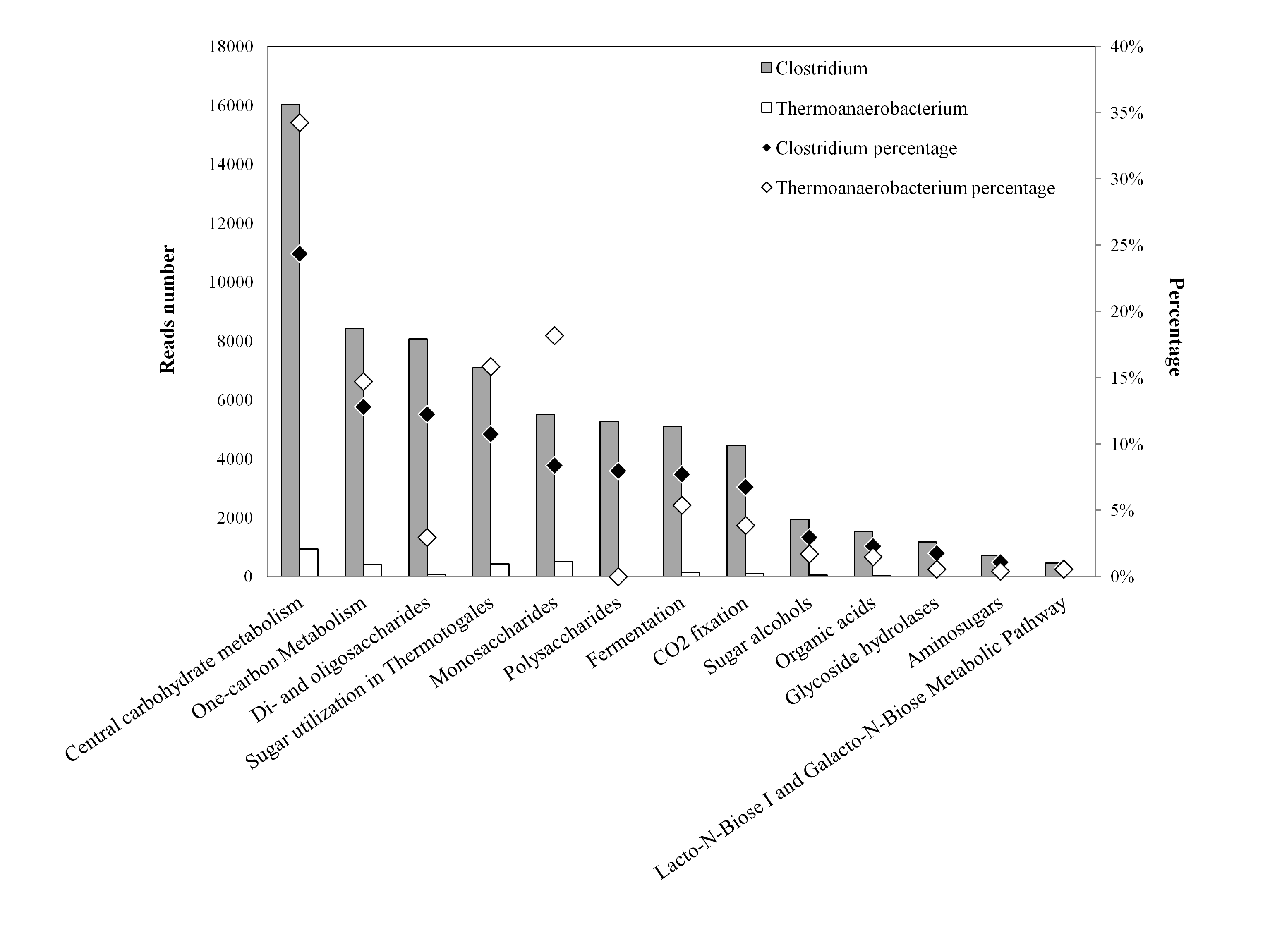


Figure S6 Relative distribution of different metabolism subsystems of genus *Clostridium* and *Thermoanaerobacterium* in the enriched thermophilic cellulolytic sludge metagenome using SEED Carbohydrates Metabolism subsystems in the MG-RAST server.
