# Supplementary material for: Mining of Novel Thermo-Stable Cellulolytic Genes from a Thermophilic Cellulose-Degrading Consortium by Metagenomics
Source: PLoS One. 2013 Jan 14;8(1):e53779. doi: 10.1371/journal.pone.0053779 (PMC3544849; doi:10.1371/journal.pone.0053779)
Supplement: Figure S7 — Gel analysis results of the predicted putative genes. Gene size of each band was shown in unit of amino acids. The incorrect gene size was marked in red frame. (DOC) [file pone.0053779.s007.doc]

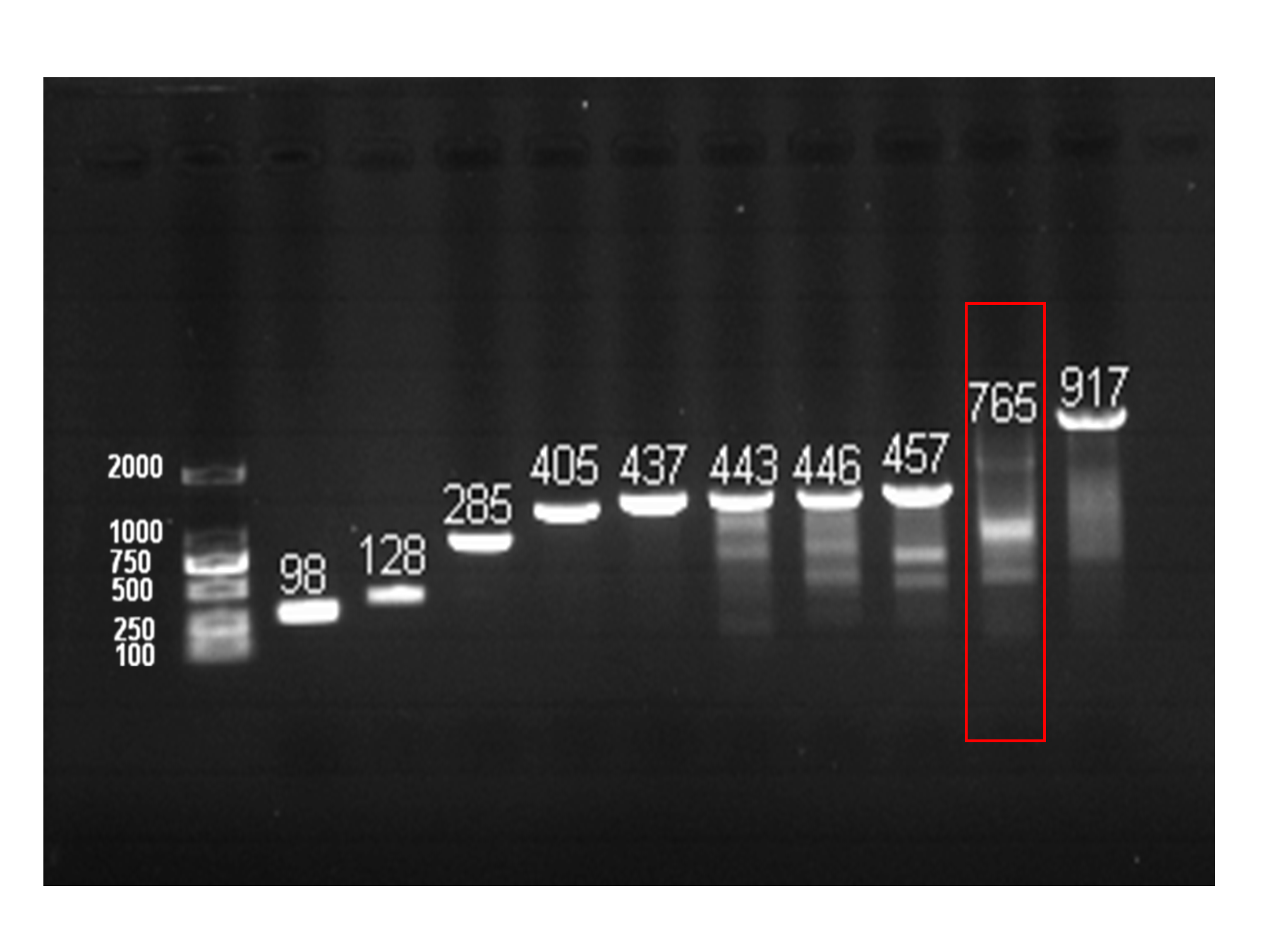


Figure S7 Gel analysis results of the predicted putative genes. Gene size of each band was shown in unit of amino acids. The incorrect gene size was marked in red frame.
