# Supplementary material for: Mining of Novel Thermo-Stable Cellulolytic Genes from a Thermophilic Cellulose-Degrading Consortium by Metagenomics
Source: PLoS One. 2013 Jan 14;8(1):e53779. doi: 10.1371/journal.pone.0053779 (PMC3544849; doi:10.1371/journal.pone.0053779)
Supplement: Table S1 — Velvet assembly statistics. (DOC) [file pone.0053779.s008.doc]

Table S1 Velvet assembly statistics

|  | Total contigs | Contigs  >300 bp | Contigs  >1 kb | Contigs  >50 kb | Contigs  >100 kb |
| --- | --- | --- | --- | --- | --- |
| Total Bases [Mb] | 40.0 | 38.6 | 28.5 | 2.7 | 0.8 |
| Number of contigs | 52579 | 23211 | 7630 | 34 | 6 |
| N50 [bp] | 2956 | 3772 | 1141 | 78312 | 189044 |
| largest contig [bp] | 202468 | 202468 | 202468 | 202468 | 202468 |
