# Supplementary material for: Mining of Novel Thermo-Stable Cellulolytic Genes from a Thermophilic Cellulose-Degrading Consortium by Metagenomics
Source: PLoS One. 2013 Jan 14;8(1):e53779. doi: 10.1371/journal.pone.0053779 (PMC3544849; doi:10.1371/journal.pone.0053779)
Supplement: Table S2 — Properties of the 10 predicted carbohydrate-active enzyme candidates tested for assembly authority. (DOC) [file pone.0053779.s009.doc]

Table S2 Properties of the 10 predicted carbohydrate-active enzyme candidates tested for assembly authority.

| Gene ID | Length [AA] | Pfam ID | CAZy family | E-Value^1)^ | Assembly Validation | | Domain organization^3)^ |
| --- | --- | --- | --- | --- | --- | --- | --- |
|  |  |  |  |  | Identities^2)^ | Gaps |  |
| gene_id_1209 | 98 | PF00457 | GH11 | 5.20E-08 | 235/235 (100%) | 0/235 (0%) | 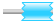**GH11** |
| gene_id_4519 | 128 | PF00942 | CBM03 | 5.10E-20 | 325/325 (100%) | 0/325 (0%) | 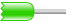**CBM03** |
| gene_id_10747 | 285 | PF00722 | GH16 | 9.80E-05 | 857/858 (99%) | 0/858 (0%) | 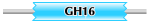 |
| gene_id_7449 | 405 | PF00722 | GH16 | 5.90E-13 | 1206/1209 (99%) | 0/1209 (0%) | 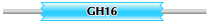 |
| gene_id_414 | 437 | PF00232 | GH01 | 2.40E-84 | 1296/1298 (99%) | 0/1298 (0%) | 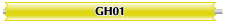 |
| gene_id_19651 | 443 | PF00232 | GH01 | 4.40E-169 | 1227/1227 (100%) | 0/1227 (0%) | 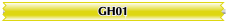 |
| gene_id_29064 | 446 | PF00232 | GH01 | 2.20E-154 | 1227/1238 (99%) | 0/1238 (0%) | 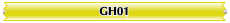 |
| gene_id_9786 | 457 | PF00232 | GH01 | 6.00E-167 | 1273/1273 (100%) | 0/1273 (0%) | 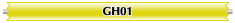 |
| gene_id_18718^4)^ | 765 | PF00759 | GH09 | 6.90E-06 | NA | NA | 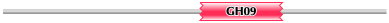 |
| gene_id_9429 | 917 | PF00933 | GH03 | 1.40E-27 | 2640/2640 (100%) | 0/2640 (0%) | 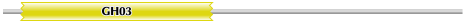 |

- - - 1. Based on Pfam_scan hit to PfamA database.
      2. Identities defined as the length of insert aligned/total base of the predicted gene (number in percentage), NA: the target gene cannot be correctly inserted.
      3. Domains are color-coded based on identified GH family and function associated with identified GH family: **blue** (endohemicellulases): GH16, GH11; **red** (Endoglucanases or Exoglucanases): GH09; **yellow** (oligosaccharide degrading enzymes): GH03, GH01; **green (CBM)**: CBM03.
      4. ORFs cannot be amplified with specifically designed primer.
