# Supplementary material for: Mining of Novel Thermo-Stable Cellulolytic Genes from a Thermophilic Cellulose-Degrading Consortium by Metagenomics
Source: PLoS One. 2013 Jan 14;8(1):e53779. doi: 10.1371/journal.pone.0053779 (PMC3544849; doi:10.1371/journal.pone.0053779)
Supplement: Table S3 — Glycoside hydrolases from the enriched thermophilic cellulolytic culture. (DOC) [file pone.0053779.s010.doc]

Table S3. Glycoside hydrolases from the enriched thermophilic cellulolytic culture

| GH family^1)^ | Pfam Model^2)^ | Gene Count^3)^ |  | GH family^1)^ | Pfam Model^2)^ | Gene Count^3)^ |
| --- | --- | --- | --- | --- | --- | --- |
| GH1^4)^ | PF00232 | 12 |  | GH38 | PF01074 | 10 |
| GH2 | PF00703 | 23 |  | GH39 | PF01229 | 3 |
| GH3^4)^ | PF00933 | 24 |  | GH42 | PF02449 | 12 |
| GH4 | PF02056 | 14 |  | GH45^4)^ | PF02015 | 0 |
| GH5^4)^ | PF00150 | 0 |  | GH46 | PF01374 | 0 |
| GH6^4)^ | PF01341 | 0 |  | GH47 | PF01532 | 0 |
| GH7^4)^ | PF00840 | 0 |  | GH48^4)^ | PF02011 | 2 |
| GH8^4)^ | PF01270 | 2 |  | GH49 | PF03718 | 0 |
| GH9^4)^ | PF00759 | 19 |  | GH52 | PF03512 | 1 |
| GH10 | PF00331 | 12 |  | GH56 | PF01630 | 0 |
| GH11 | PF00457 | 9 |  | GH57 | PF03065 | 12 |
| GH12^4)^ | PF01670 | 0 |  | GH59 | PF02057 | 0 |
| GH13 | PF00128 | 0 |  | GH61 | PF03443 | 0 |
| GH14 | PF01373 | 0 |  | GH62 | PF03664 | 0 |
| GH15 | PF00732 | 6 |  | GH63 | PF03200 | 1 |
| GH16 | PF00722 | 9 |  | GH65 | PF03632 | 0 |
| GH17 | PF00332 | 0 |  | GH67 | PF03648 | 0 |
| GH18 | PF00704 | 8 |  | GH68 | PF02435 | 0 |
| GH19 | PF00182 | 1 |  | GH70 | PF02324 | 0 |
| GH20 | PF00728 | 12 |  | GH71 | PF03659 | 0 |
| GH22 | PF00062 | 0 |  | GH72 | PF03198 | 0 |
| GH24,GH104^5)^ | PF00959 | 0 |  | GH73 | PF01832 | 0 |
| GH25 | PF01183 | 1 |  | GH75 | PF07335 | 0 |
| GH26 | PF02156 | 4 |  | GH76 | PF03663 | 3 |
| GH27 | PF02065 | 0 |  | GH77 | PF02446 | 7 |
| GH28 | PF00295 | 3 |  | GH78 | PF05592 | 0 |
| GH29 | PF01120 | 0 |  | GH79 | PF03662 | 0 |
| GH30^4)^ | PF02055 | 5 |  | GH81 | PF03639 | 1 |
| GH31 | PF01055 | 14 |  | GH83 | PF00423 | 0 |
| GH32 | PF00251 | 0 |  | GH85 | PF03644 | 0 |
| GH33 | PF02012 | 0 |  | GH88 | PF07470 | 3 |
| GH34 | PF00064 | 0 |  | GH89 | PF05089 | 0 |
| GH35 | PF01301 | 3 |  | GH100 | PF12899 | 0 |
| GH37 | PF01204 | 0 |  | GH102 | PF03562 | 0 |

1. Glycoside Hydrolase (GH) family classification according to the CAZy database. GH families without Pfam model are not presented.
2. Pfam model associated with the respective GH family.
3. Number of ORFs containing the domain for a particular GH family.
4. GH families associated with cellulase.
5. GH families which cannot be distinguished by Pfam model were combined
