# Supplementary material for: Mining of Novel Thermo-Stable Cellulolytic Genes from a Thermophilic Cellulose-Degrading Consortium by Metagenomics
Source: PLoS One. 2013 Jan 14;8(1):e53779. doi: 10.1371/journal.pone.0053779 (PMC3544849; doi:10.1371/journal.pone.0053779)
Supplement: Table S4 — Carbohydrate binding modules from enriched thermophilic cellulolytic culture. (DOC) [file pone.0053779.s011.doc]

Table S4 Carbohydrate binding modules from enriched thermophilic cellulolytic culture

| GH family^1)^ | Pfam Model^2)^ | Gene Count^3)^ |  | GH family^1)^ | Pfam Model^2)^ | Gene Count^3)^ |
| --- | --- | --- | --- | --- | --- | --- |
| CBM1 | PF00734 | 0 |  | CBM18 | PF00187 | 0 |
| CBM2 | PF00553 | 3 |  | CBM19 | PF03427 | 0 |
| CBM3 | PF00942 | 13 |  | CBM20 | PF00686 | 2 |
| CBM4,CBM9,CBM16,CBM22^4)^ | PF02018 | 0 |  | CBM21 | PF03370 | 0 |
| CBM5,CBM12^4)^ | PF02839 | 0 |  | CBM25 | PF03423 | 3 |
| CBM6 | [PF03422](http://pfam.sanger.ac.uk/family/PF03422) | 13 |  | CBM32 | PF00754 | 0 |
| CBM10 | PF02013 | 0 |  | CBM33 | PF03067 | 0 |
| CBM13 | PF00652 | 0 |  | CBM34 | PF02903 | 0 |
| CBM14 | PF01607 | 0 |  | CBM40 | PF02973 | 0 |
| CBM15 | PF03426 | 0 |  | CBM50 | PF01476 | 0 |
| CBM17,CBM28^4)^ | PF03424 | 0 |  | CBM51 | PF08305 | 0 |

1. Carbohydrate binding module (CBM) family classification according to the CAZy database. CBM families without Pfam model are not presented.
2. Pfam model associated with the respective CBM family.
3. Number of ORF containing the domain for a particular CBM family.
4. CBM families which cannot be distinguished by Pfam model were combined.
