# Supplementary material for: Mining of Novel Thermo-Stable Cellulolytic Genes from a Thermophilic Cellulose-Degrading Consortium by Metagenomics
Source: PLoS One. 2013 Jan 14;8(1):e53779. doi: 10.1371/journal.pone.0053779 (PMC3544849; doi:10.1371/journal.pone.0053779)
Supplement: Table S5 — Comparison between metagenomic study of cow rumen microbes (10) and this study. (DOC) [file pone.0053779.s012.doc]

Table S5 Comparison between metagenomic study of cow rumen microbes (10) and this study

| **Item** | **Hess et al (Science, 2011)** | **This Study** |
| --- | --- | --- |
| Microbes | cow rumen microbes adherent to switchgrass | pre-enriched thermophilic cellulolytic consortia |
| Approximated complexity | 1000 OTUs in total | top 4 OTUs taken 84% population |
| Dataset size (base pairs) | 267.9 Giga | 1.2 Giga |
| Kmer coverage | 30× | 14× |
| Sequences usage in assembly | 65% | 72% |
| Assembly accuracy determined by size | 68% | 90% |
| ORFs defined | 2,547,270 | 31,499 |
| ORFs with GH or CBM | 27,755  (1.1% of all ORFs) | 253  (0.8% of all ORFs) |
